# Does the “high sugar” trait of perennial ryegrass cultivars express under temperate climate conditions?

**DOI:** 10.1111/gfs.12406

**Published:** 2019-01-28

**Authors:** M. Jordana Rivero, Oscar A. Balocchi, Cristian J. Moscoso, Juan Agustín Siebald, Fabián Lukas Neumann, Don Meyer, Michael R. F. Lee

**Affiliations:** ^1^ Departamento de Ciencias Agropecuarias y Acuícolas Facultad de Recursos Naturales Universidad Católica de Temuco Temuco Chile; ^2^ Rothamsted Research Okehampton UK; ^3^ Facultad de Ciencias Agrarias Instituto de Producción Animal Universidad Austral de Chile Valdivia Chile; ^4^ Instituto de Investigaciones Agropecuarias INIA Remehue Osorno Chile; ^5^ CIA‐CENEREMA Facultad de Ciencias Veterinarias Universidad Austral de Chile Valdivia Chile; ^6^ Rock River Laboratory Inc Watertown Wisconsin; ^7^ Bristol Veterinary School University of Bristol Langford UK

**Keywords:** defoliation frequency, G  ×  E interaction, high sugar grass, temperate climate, water‐soluble carbohydrates

## Abstract

The objective was to evaluate water‐soluble carbohydrate (WSC) and crude protein (CP) concentration of perennial ryegrass (PRG) cultivars with different genetic potential for producing WSC under two contrasting agronomic managements in temperate climate (southern Chile). A 4 × 2 factorial design was randomly allocated to 24 plots (31 m^2^ each, three blocks): four PRG cultivars (diploid standard cultivar, “2nSt”; tetraploid standard cultivar, “4nSt”; diploid high sugar cultivar developed in New Zealand, “2nHSNZ”; and tetraploid high sugar cultivar developed in Europe, “4nHSEU”) and two agronomic managements (“favourable,” defoliations at three leaves per tiller and nitrogen (N) fertilization rate of 83.3 kg N ha^−1 ^year^−1^; “unfavourable,” defoliations at two leaves per tiller and N fertilization rate of 250 kg N ha^−1 ^year^−1^). Herbage samples were collected in early spring, spring, summer and autumn. Concentration of WSC did not differ among cultivars in spring and summer, averaging 194 and 251 g/kg DM, respectively. The cultivar 4nHSEU had the greatest WSC concentration in early spring and autumn (187 and 266 g/kg DM, respectively) and the greatest CP concentration across samplings (average 230 g/kg DM). Favourable management improved WSC concentrations in early spring and summer and decreased CP in spring, summer and autumn. Annual DM yield did not vary with cultivar or management, averaging 8.43 t/ha. Within a 12‐month study at one site in a temperate environment in southern Chile, PRG cultivars have not shown a consistent expression of the “high sugar” trait, where a genetic × environment interaction might be operating.

## 1 INTRODUCTION

Perennial ryegrass (*Lolium perenne* L.) is the forage species most widely used in temperate pastures (Wilkins & Humphreys, 2003) as it grows well in a wide range of soil fertility situations, is easy to establish and manage (Charlton & Stewart, 1999), with high forage yields, digestibility and adaptation to a range of practices (McDonagh, O'Donovan, McEvoy, & Gilliland, 2016). However, its growth rate and nutritional components vary greatly seasonally and with regrowth time, nitrogen (N) fertilization rate, ploidy level and cultivars. It has been widely stated that the longer the regrowth period, i.e., the greater the number of leaves per tiller (Fulkerson, Slack, Hennessy, & Hough, 1998) and/or the greater the ploidy level (Gilliland, Barrett, Mann, Agnew, & Fearon, 2002) the higher the water‐soluble carbohydrate (WSC) concentration and that the greater the N fertilization the higher the crude protein (CP) content at the expense of WSC in the grass (Tas et al., 2006). However, the heading date has not shown a consistent effect on WSC content of PRG cultivars (Gilliland et al., 2002). Crude protein and WSC contents vary markedly with season, with CP peaks during autumn and early spring (Roche et al., 2009), whereas WSC accumulate throughout summer and autumn, leading to the highest total accumulated during winter and minimum values during early spring (Pollock & Jones, 1979). These variations in nutritive components throughout the year lead to an imbalance between readily available energy, in the form of WSC, and nitrogen in the rumen.

In relation to cultivar differences, in the last few decades, some UK and European grass breeding programmes have focused on increasing average WSC, developing “high sugar grasses” (HSG; Humphreys, 1989). The final objective pursued was driven by improving nutritional quality for grazing ruminants. It was hypothesized that synchronizing energy release from WSC and non‐protein nitrogen (ammonia) from plant breakdown in the rumen would optimize rumen microbial protein synthesis, decrease ruminal ammonia excess and subsequently improve ruminal nitrogen utilization (Lee et al., 2003). This would lead to a greater nitrogen use efficiency (NUE), reducing N excreted through urea and improve animal performance (Edwards, Parsons, Rasmussen, & Bryant, 2007). A recent review has summarized the potential of WSC levels in pasture in relation to animal production (Lee, Rivero, & Cone, 2018). Lower amounts of urea N deposited on soil as urine would also have an environmental benefit through reducing nitrous oxide emissions (a potent greenhouse gas) and lower nitrate leaching (a major component of emissions into water courses; Rasmussen, Parsons, Xue, & Newman, 2009).

Nevertheless, the extent and consistency of expression of the “high sugar” trait have been related with the environment in which the HSG has been tested. This trait is reported to be expressed consistently in HSG cultivars in Northern Europe (Norway, Denmark, Sweden) and UK (Halling, Longland, Martens, Nesheim, & O'Kiely, 2004). However, HSG cultivars under New Zealand conditions have shown variable results (Hume, Hickey, Lyons, & Baird, 2010; Parsons et al., 2004). In fact, according to Parsons, Edwards, et al. (2011), an environment by genetic interaction may have been operating. These authors comparing UK with New Zealand conditions found that although the UK is on average only 2°C cooler, temperatures in spring are 5 to 7°C lower in the UK around the equinox, and this follows a much colder and longer winter. This difference in environmental conditions has led New Zealand grass breeders to start developing local HSG cultivars more adapted to their particular environment, as is the case for the recently released cultivar “Expo” by PGG Wrightson Ltd. (Rasmussen et al., 2009).

In the last decade, some HSG cultivars have been introduced to South America, e.g., Chile, with the aim of improving pasture quality and animal productivity. To the best of our knowledge, there is only one trial that has investigated WSC and N within PRG cultivars in South America (Moscoso & Balocchi, 2016). They investigated two cultivars bred for “high sugar” (AberAvon and AberDart AR1) and two standard cultivars (Jumbo and Arrow AR1) all diploids. The authors found no difference between cultivars when assessing the WSC and N by NIR from oven‐dried samples of pasture. However, they found some trend towards increased WSC in the base of tillers in the HSG cultivars compared with the standard cultivars, although that trend was not consistent among sampling periods (from early spring to summer).

According to Peel, Finlayson, and McMahon (2007), New Zealand (North and South islands, from 34°40′S to 47°00′S) and southern Chile (specifically Los Ríos and Los Lagos regions, from 39°15′S to 44°14′S) have similar climate, classified as “marine—mild winter,” a temperate climate without dry season and with a warm summer (“Cfb” climate class). Given these similarities in climate conditions, it was hypothesized that a PRG cultivar that expresses the HSG trait under New Zealand environmental conditions could express more consistently the trait in southern Chile compared with a European HSG cultivar during critical seasons, i.e., autumn and spring. Moreover, with the aim of augmenting differences in WSC and CP concentrations, cultivars were submitted to contrasting regimes, managing together N fertilization rate and defoliation frequency.

Therefore, the objective of this study was to evaluate nutritional composition, emphasizing WSC and CP concentration, of PRG cultivars with different genetic potential for producing WSC under two contrasting agronomic managements in southern Chile.

## 2 MATERIALS AND METHODS

### 2.1 Experimental site

**Figure 1:**
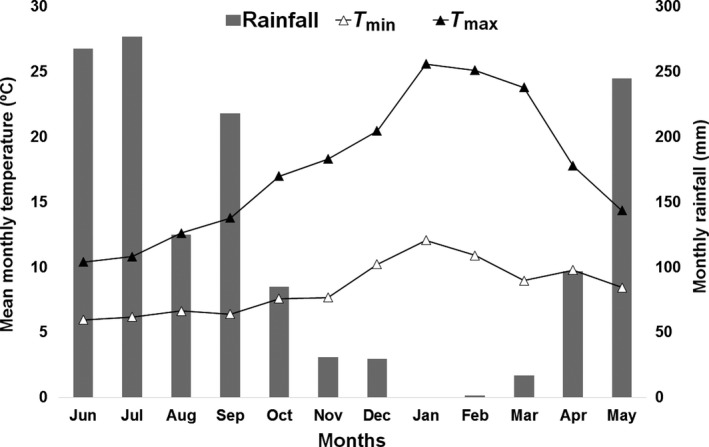
Monthly rainfall and mean monthly minimum and maximum temperatures from June 2014 to May 2015 in the experimental site (Valdivia, Los Rios Region—southern Chile)

The study was carried out between June 2014 and May 2015 at the research station of the Universidad Austral de Chile, Valdivia, Chile (39°47′S, 72°12′W). This climate is characterized by a high relative humidity, low temperatures and high annual rainfall record, which has a minimum noticeable in summer, although without a dry season. Mean daily temperature and rainfall for the experimental period are shown in Figure 1. Weather conditions of southern Chile when this study was carried out were characterized by average daily maximum temperatures ranging from 10.4 to 25.6°C (average 17.5°C), and average daily minimum temperatures ranging between 6.0 and 12.1°C (average 8.4°C).

Due to an unexpected drought during summer (January to March 2015), and with the objective of preserving the pasture, plots were sprinkler irrigated, during the afternoon, with 30 mm of water when soil water tension, measured with a tensiometer (Irrometer model R, Irrometer Company, Riverside, CA, USA), reached 40 kPa.

### 2.2 Treatments

Plots of pasture were established in March 2014 on an experimental area of 744 m^2^ (divided into three blocks of 8 plots each, and each plot was 31 m^2^). In each block, eight treatments were allocated randomly. Treatments corresponded to a factorial combination of four PRG cultivars: diploid standard cultivar (intermediate heading date: “2nSt”); tetraploid standard cultivar (late heading date: “4nSt”); diploid HSG cultivar developed in New Zealand (late heading date: “2nHSNZ”); and a tetraploid HSG cultivar developed in Europe (intermediate heading date: “4nHSEU”), and two contrasting agronomic managements with regard to their expected effects on WSC concentration “favourable” regime (consisting of defoliations at the stage of three leaves per tiller and with a N fertilization rate equivalent to 83.3 kg N ha^−1 ^year^−1^) and a “unfavourable” regime (consisting of defoliations at the stage of two leaves per tiller and with a N fertilization rate equivalent to 250 kg N ha^−1 ^year^−1^).

### 2.3 Pasture management

Prior to sowing, and following soil tests, the experimental site was fertilized with 130 kg of P_2_O_5_ per ha, 100 kg of K_2_O per ha and 2000 kg CaCO_3_ per ha, to ensure suitable level of key nutrients (P‐Olsen > 12 mg/kg, K > 0.35 cmol/kg) and an adequate pH (6 to 6.5). A full soil test was performed during the experimental period, and corrective fertilization was realized in autumn to maintain suitable level of key nutrients (30 kg of P_2_O_5_ per ha, 66 kg of K_2_O per ha). After sowing in March 2014 (target: 1200 viable seeds/m^2^), a first cut of homogenization was performed to all plots in June 2014 with a rotary lawnmower to a height of 4 cm above ground level. After that, the N fertilization was commenced. For both management groups (annual rate of 83 or 250 kg N per ha), the doses of N fertilizer were partitioned in monthly allocations throughout the experimental period. Partial doses were determined from the N extraction of PRG pastures in the same site during previous years as proposed by Loaiza, Balocchi, and Bertrand (2017). Thus, each annual N fertilizer rate was divided into successive monthly applications across 12 months. The source of N in the fertilizer used was ammonium nitrate (27% N).

Subsequently, pasture sampling was carried out each time the plots averaged two or three leaves per tiller (depending on the management factor) and all plots of the same management were harvested in the same day. On average, swards defoliated at two leaves per tiller were sampled every 29 days (ranging from 18 to 55 days) and at three leaves per tiller every 51 days (ranging from 32 to 87 days).

### 2.4 Herbage sampling

To determine the time of sampling, leaf stage was checked periodically by assessing the number of leaves per tiller (*n* = 10) from each plot. Prior to harvest fresh material, the edges of each plot to be sampled were removed with a lawnmower (strip of 83 cm width). At each sampling time, fresh grass pooled samples of approximately 150 g per plot were taken, using hand shear to a simulated grazing height of 5 cm. Sampling was achieved, block by block, within a short window around noon (Easton, Stewart, Lyons, Parris, & Charrier, 2009; Parsons et al., 2004) to avoid complications due to diurnal patterns of WSC metabolism, frozen immediately in liquid N_2_ on site to prevent WSC wastage post‐harvest, stored at −20°C until freeze dried and then ground through a 1 mm sieve.

After sampling, the remaining area of grass (after the edges removal) was measured and all remaining herbage of each plot was removed by harvesting all forage above 5 cm, as suggested by Fulkerson, Slack, and Lowe (1994), to optimize ryegrass growth and persistence. Harvested material was weighed fresh and homogenized to collect a subsample of approximately 10% of the total forage collected. This subsample was then weighed fresh and oven‐dried for 48–72 hr to obtain the percentage of DM. This percentage DM was used to calculate the total DM amount harvested from each plot (edges not included) in each cut, expressed as t/ha. All the samplings performed during one calendar year (after the homogenization cut in June 2014) were added together to calculate the total annual DM production in the establishment year.

### 2.5 Analytical procedures

The herbage collected from samplings of early spring (August 2014), spring (October 2014), summer (January 2015) and autumn (April 2015) was analysed for WSC (phenol–sulphuric acid assay; DuBois, Gilles, Hamilton, Rebers, & Smith, 1956), CP (Kjeldahl method, N × 6.25, AOAC, 1995), neutral detergent fibre (NDF; using sodium sulphite, Van Soest, Robertson, & Lewis, 1991), acid detergent fibre (ADF; Van Soest et al., 1991), ash (AOAC, 1995) and digestible organic matter on dry‐matter basis (DOMD; Tilley & Terry, 1963). Metabolizable energy (ME) was calculated from DOMD using the following equation: ME (Mcal per kg DM) = DOMD × 0.016 (McDonald et al., 2011).

### 2.6 Statistical analysis

Data regarding WSC concentration, WSC to CP ratio and the other nutrients over time were analysed by repeated measures analysis of variance (ANOVA) for a completely randomized block design with a factorial arrangement of treatments. These analyses allowed for comparison of cultivar and management means across time and their interactions. The sampling times reported here were defined as early spring (August sampling), spring (October sampling), summer (January sampling) and autumn (April sampling). The model used for the ANOVA was as follows:

$$Y_{\mathrm{hijkl}}=\mu +b_{h}+s_{i}+\alpha_{j}+\beta_{k}+\pi_{l}+{\left(\alpha \beta \right)}_{\mathrm{jk}}+{\left(\alpha \pi \right)}_{\mathrm{jl}}+{\left(\beta \pi \right)}_{\mathrm{kl}}+{\left(\alpha \beta \pi \right)}_{\mathrm{jkl}}+\varepsilon_{\mathrm{hijkl}}$$

where *b* is block effect, *s* is subject effect, α, β and π are management, cultivar and date effects, respectively, followed by their double and triple interactions and the random error. This procedure applies an adjustment to the degrees of freedom (*df*) for testing the effects, given by the *df* correction factor. Fisher least significant difference (LSD) was used for the statistical separation of means.

Total annual DM yield and DM harvested per cut from each of the eight factorial treatments (four cultivars × two managements) were compared by a two‐way ANOVA, which model is as follows:

$$Y_{\mathrm{hijkl}}=\mu +b_{h}+\alpha_{i}+\beta_{j}+{\left(\alpha \beta \right)}_{\mathrm{ij}}+\varepsilon_{\mathrm{hij}}$$

where *b* is block effect, α and β are management and cultivar, respectively, followed by their interaction and the random error.

Single correlations were calculated among WS, CP, NDF, ADF and ME to assess the relationship among individual nutrients. A multiple regression was calculated with WSC as the response variable and CP and NDF as the predictor to assess the relationship among main nutrients. Additionally, single and multiple regression were performed by group, i.e., management and cultivar, to assess the whether these relationships vary between managements or genotypes.

The Genstat^®^ 18th (©VSN International Ltd., UK) statistical system was used for the analysis.

## 3 RESULTS

### 3.1 Water‐soluble carbohydrates and crude protein concentrations

**Figure 2:**
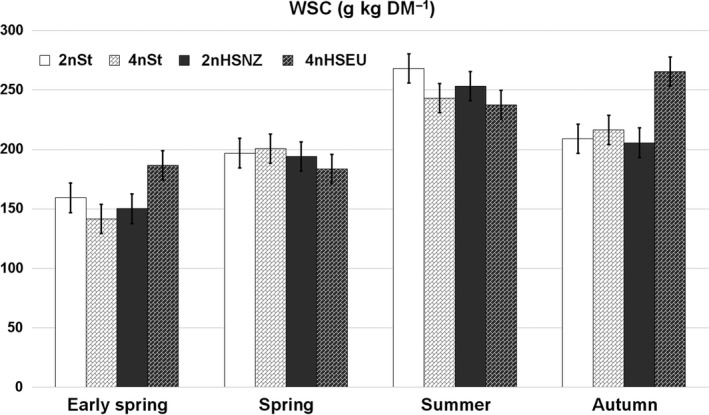
Water‐soluble carbohydrates (WSC) of four cultivars of perennial ryegrass^†^ varying in ploidy level and potential production of water‐soluble carbohydrates across four sampling periods (Early Spring, Spring, Summer and Autumn). ^†^2n: diploid, 4n: tetraploid, St standard cultivar, HS high sugar cultivar, NZ: origin New Zealand, EU: origin Europe

Concentration of WSC was not different among cultivars within sampling time for spring and summer, averaging 194 and 251 g/kg DM, respectively. In early spring, 4nHSEU had greater WSC than the 4nSt cultivar and was similar to the diploid cultivars (Figure 2). In autumn, 4nHSEU cultivar had significantly greater WSC concentration than the remaining three cultivars (266 g/kg DM for 4nHSEU vv. 211 g/kg DM as an average of the other three cultivars; Table 1 and Figure 2). Regarding management, a significant effect was found in early spring and summer, with the favourable treatment contained more WSC than the unfavourable group, particularly in the summer when the difference was 84.1 g/kg DM (Table 2).

**Table 1:** ANOVA summary of the nutritional composition of leaves of four cultivars under two contrasting managements sampled in four different times of the year

	WSC	WSC:CP	CP	ME	NDF	ADF
Cultivar (C)	0.088	0.183	**0.002**	**0.046**	**<0.001**	**<0.001**
Management (M)	**<0.001**	**<0.001**	**<0.001**	0.555	0.840	0.677
C × M	0.089	0.185	0.118	0.429	0.264	0.259
Time (T)	**<0.001**	**<0.001**	**<0.001**	**0.018**	**<0.001**	**<0.001**
T × C	**0.036**	0.287	0.806	0.541	**0.035**	**0.002**
T × M	**<0.001**	**<0.001**	**<0.001**	0.758	**<0.001**	**<0.001**
T × C × M	0.843	0.933	0.819	0.526	0.622	0.136
*df* correction factor	0.6849	0.6965	0.8479	0.8365	0.7615	0.7237

ADF: acid detergent fibre; CP: crude protein; *df*: degrees of freedom; ME: metabolizable energy; NDF: neutral detergent fibre; WSC: water‐soluble carbohydrates. Values in bold highlights significant effects.

**Table 2:** Water‐soluble carbohydrates (WSC) and crude protein (CP) concentrations, and the WSC to CP ratio of perennial ryegrass pastures under contrasting managements (M) regarding their effect on WSC concentration across four sampling periods (T) (Early Spring, Spring, Summer and Autumn)

	Early spring	Spring	Summer	Autumn	Early spring	Spring	Summer	Autumn	Early spring	Spring	Summer	Autumn
	WSC (g/kg DM)				CP (g/kg DM)				WSC:CP			
Unfavourable^a^	145.8	183.1	208.5	230.7	291.8	221.9	179.7	262.6	0.50	0.85	1.23	0.88
Favourable^b^	173.1	204.5	292.6	217.5	298.5	173.2	123.9	186.8	0.58	1.22	2.40	1.17
LSD (*p* < 0.05)	28.03 within management comparisons				17.79 within management comparisons				0.270 within management comparisons			
	26.49 between management comparisons				17.12 between management comparisons				0.254 between management comparisons			
*p* value for M × T	<0.001				<0.001				<0.001			

^a^Unfavourable: annual N fertilization rate of 250 kg/ha and defoliated at the stage of 2 leaves per tiller. ^b^Favourable: annual N fertilization rate of 83.3 kg/ha and defoliated at the stage of 3 leaves per tiller.

**Figure 3:**
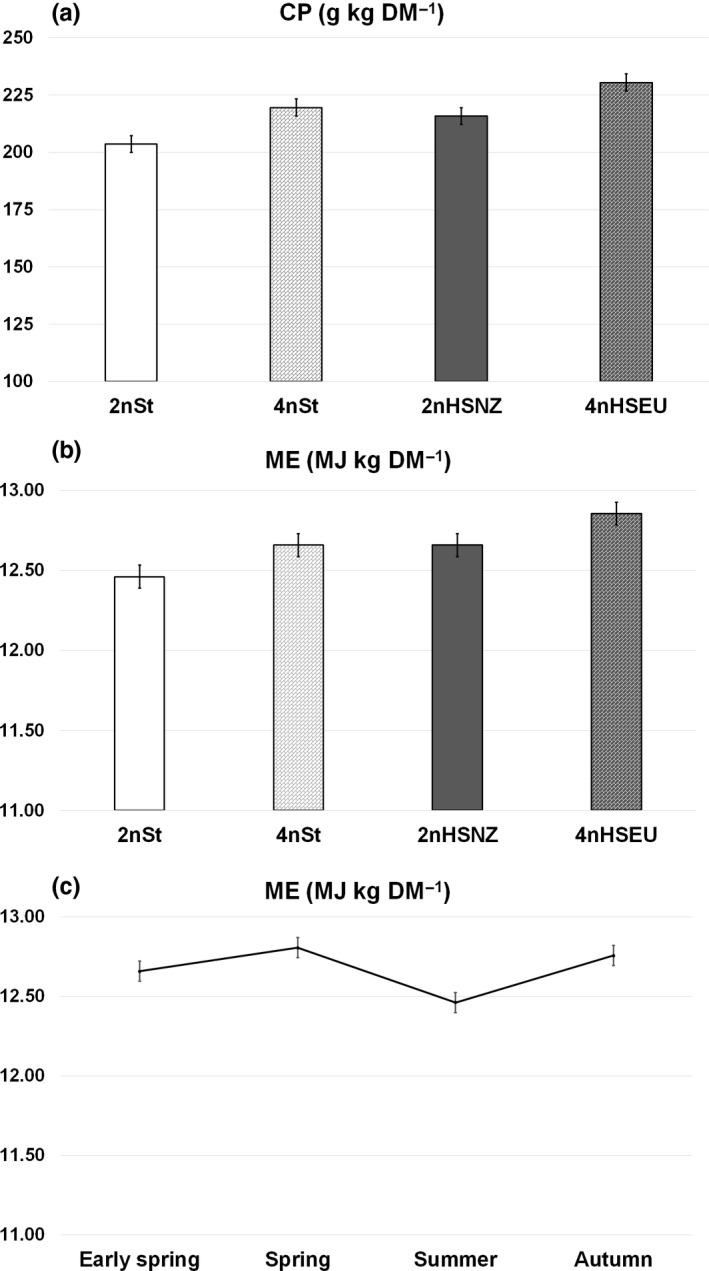
Crude protein (a) and metabolizable energy^†^ (b) of four cultivars^‡^ of perennial ryegrass varying in ploidy level and potential production of water‐soluble carbohydrate (WSC), and metabolizable energy (c) as an average of the four cultivars across four sampling periods (Early Spring, Spring, Summer and Autumn).^†^ CP: crude protein: ME: metabolizable energy. ^‡^2n: diploid, 4n: tetraploid, St standard cultivar, HS high sugar cultivar, NZ: origin New Zealand, EU: origin Europe

Crude protein varied among cultivars; 4nHSEU had the greatest value (230 g/kg DM), 4nSt and 2nHSNZ an intermediate value (average 218 g/kg DM) and the 2nSt the lowest value (204 g/kg DM, Figure 3a). Crude protein concentration was similar between managements in early spring, averaging 295 g/kg DM. However, unfavourable management plots showed greater CP concentrations for the remaining season, surpassing the favourable management by between 48.7 and 75.8 g/kg DM (Table 2).

Ratio between WSC and CP was similar among cultivars, ranging from 1.21 for the 2nSt cultivar to 1.07–1.08 for the remaining cultivars (LSD = 0.147; *p* < 0.05). The ratio was the lowest in early spring and was similar between managements, averaging 0.54. Then, it increased until summer and decreased in autumn, while the favourable management significantly improved that ratio through the seasons (Table 2).

### 3.2 Energy and fibre concentrations

Metabolizable energy concentration of grass leaves was the highest in 4nHSEU cultivar (12.85 MJ/kg DM) and the lowest for 2nSt cultivar (12.46 MJ/kg DM; *p* < 0.05), and both were not statistically different than the intermediate values observed in 4nSt and 2nHSNZ cultivars (12.66 MJ/kg DM each; Figure 3b). Metabolizable energy concentration was significantly lower during the summer (12.46 MJ/kg DM) than the other seasons which did not differ among each other (average 12.74 MJ/kg DM, Figure 3c).

Concentration of NDF was the lowest in early spring and the highest in summer with different trends among cultivars along all periods (Table 3). In early spring, the 4nHSEU cultivar had the lowest NDF concentration, followed by the 4nSt cultivar which was similar to the 2nSt cultivar. The highest NDF concentration was observed in the 2nHSNZ cultivar which was similar to the 2nSt cultivar (Table 3). During spring, the diploid cultivars had the highest values (average 386 g/kg DM) and the tetraploid cultivars the lowest (average 364 g/kg DM), although tetraploid cultivars and the 2nHSNZ were similar (Table 3). However, during summer, there was no difference between cultivars in NDF, averaging 392 g/kg DM. During autumn, the tetraploid cultivars had the lowest values (average 364 g/kg DM) and the diploids the highest (average 384 g/kg DM). However, 4nSt did not differ from the diploid cultivars (Table 3). Comparing managements, significant differences were observed in early spring and autumn sampling, with the favourable treatment having 37.7 g/kg DM more NDF than the unfavourable management (Table 3).

**Table 3:** Neutral detergent fibre (NDF) and acid detergent fibre (ADF) of four cultivars of perennial ryegrass varying in ploidy level and potential production of water‐soluble carbohydrate (WSC), and under contrasting managements regarding the effect on WSC concentration, across four sampling periods (Early Spring, Spring, Summer and Autumn)

	Early spring	Spring	Summer	Autumn	Early spring	Spring	Summer	Autumn
	NDF (g/kg DM)				ADF (g/kg DM)			
Cultivar^a^								
2n	335.5	394.2	390.6	385.3	199.2	240.5	216.2	221.2
4nSt	323.8	363.9	389.5	368.3	196.3	223.1	229.1	218.3
2nHSNZ	349.8	377.5	401.5	382.3	205.0	224.9	222.4	222.1
4nHSEU	290.4	364.6	385.2	360.2	170.3	223.4	216.3	205.9
LSD (*p* < 0.05)	21.24 within cultivar comparisons				11.90 within cultivar comparisons			
	21.47 between cultivar comparisons				11.97 between cultivar comparisons			
Management^b^								
Unfavourable	306.0	369.2	396.3	354.9	183.6	225.9	220.4	208.6
Favourable	343.7	380.9	387.1	393.2	201.8	230.0	221.6	225.1
LSD (*p* < 0.05)	15.02 within management comparisons				8.42 within management comparisons			
	30.36 between management comparisons				8.46 between management comparisons			
*p* value for M × T	<0.001				<0.001			

^a^2n: diploid, 4n: tetraploid, St standard cultivar, HS high sugar cultivar, NZ: origin New Zealand, EU: origin Europe. ^b^Unfavourable: annual N fertilization rate of 250 kg/ha and defoliated at the stage of 2 leaves per tiller; Favourable: annual N fertilization rate of 83.3 kg/ha and defoliated at the stage of 3 leaves per tiller.

Concentration of ADF was lowest in early spring and highest in spring with different trends among cultivars across all periods (Table 3). Similar to NDF, 4nHSEU had the lowest ADF concentration in early spring and 2nHSNZ the highest, both being different than the remaining cultivars which averaged 198 g/kg DM. During spring, 2nSt cultivar had the greatest ADF concentration (241 g/kg DM) and was significantly different to the other cultivars which did not differ (average 224 g/kg DM). During summer, 2nSt and 4nHSEU had the lowest values (average 216 g/kg DM), 4nSt had the highest, and all these cultivars were similar to 2nHSNZ (Table 3). During autumn, the 4nHSEU cultivar had the lowest ADF concentration (206 g/kg DM) and differed significantly from the remaining three cultivars, which averaged 221 g/kg DM. As observed for NDF, significant differences between managements were recorded in early spring and autumn, with the favourable treatment containing 18.2 and 16.5 g/kg DM more ADF than the favourable group, respectively (Table 3).

### 3.3 Relationships among nutrient concentrations

**Figure 4:**
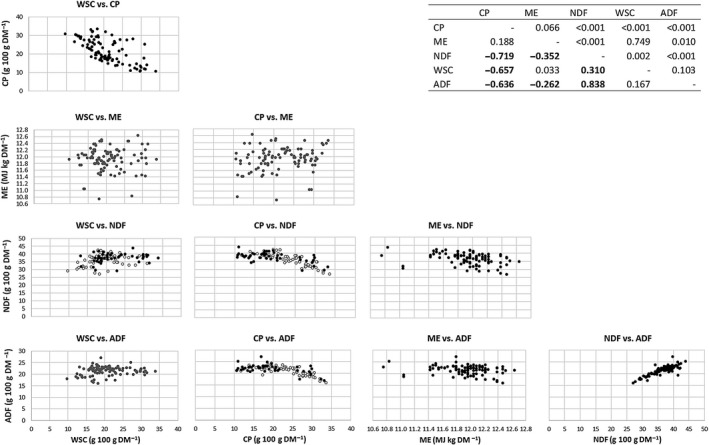
Scatter plot and correlation matrix among nutrients of perennial ryegrass pastures^†^. ^†^ Scatter plots in grey represent non‐significant correlations, when white and black circles are included in the scatter plot: ○ represents unfavourable management (annual N fertilization rate of 250 kg/ha and defoliated at the stage of 2 leaves per tiller), and ● represents favourable management (annual N fertilization rate of 83.3 kg/ha and defoliated at the stage of 3 leaves per tiller); values below the diagonal in the right‐hand top corner table are correlation coefficients and above *p* values

Scatter plot and correlation matrix among nutrients are presented in Figure 4. A negative relationship was found between CP and WSC, NDF and ADF. A weak positive correlation was found between WSC and NDF, and a weak negative correlation was found between ME and NDF. No significant correlation was found between ME and WSC concentrations. Concentration of NDF was highly and positively related to ADF as expected. No significant correlation was found between WSC and ADF, and a weak negative correlation was found between ME and ADF. Single correlations between WSC and CP showed a trend (*p* = 0.064) to vary among cultivars: −0.45 for the 4nHSEU cultivar and from −0.75 to −0.78 for the remaining cultivars. However, correlations between WSC vs. NDF, CP vs. NDF and CP vs. ADF varied between management groups, where the correlation coefficients were greater for the unfavourable group compared with the favourable group: 0.298 vs. 0.205 (WSC vs. NDF), 0.822 vs. 0.600 (CP vs. NDF) and 0.711 vs. 0.526 (CP vs. ADF), respectively.

Moreover, a multiple linear regression using CP and NDF as predictors of WSC showed a moderate fit of the model (*R* ^2^ = 0.49) and both regression coefficients were negative (WSC = 551.4 – 0.751 CP – 0.495 NDF; *p* < 0.001). Even though both coefficients were negative, the standard errors were different: 0.89 for CP and 1.57 for NDF. This multiple linear regression did not vary between managements nor among cultivars.

### 3.4 Dry‐matter production

Eight cuts were performed on the “favourable” management plots while 14 cuts were carried out for the “unfavourable” group of plots. Dry‐matter production did not vary among cultivars (*p* = 0.714) nor managements (*p* = 0.232), with not significant interaction between cultivar and management (*p* = 0.832). Total annual production averaged 8.43 t/ha, ranging from 7.75 to 8.97 t/ha. Dry matter harvested per cut did not differ among cultivars (*p* = 0.655). However, it was greater (*p* < 0.001) under the “favourable” management compared with the “unfavourable” group (1.02 v. 0.62 t/ha).

## 4 DISCUSSION

The overall aim of this study was to determine if the “high sugar” trait of PRG cultivars is expressed under temperate climatic conditions during the first year after establishment, tested in southern Chile. It was hypothesized that a cultivar bred in New Zealand for higher WSC content would have a greater chance to express the trait than a European cultivar bred for the same trait. The differences between standard and HSG cultivars were intended to be augmented by applying contrasting managements which would influence WSC and CP concentration. The results reported here comprise only one year of evaluation after the establishment of the swards and are potentially subjected to an environment × genotype × management interaction effect. Therefore, some limitations in the ability to make firm conclusions about the hypotheses are likely to be present.

### 4.1 WSC and CP concentrations among cultivars

Even though the main factor cultivar did not affect WSC concentration, the 4nHSEU surpassed the remaining cultivars on early spring and autumn. This response could be attributed to the ploidy level per se, given that Hume et al. (2010) found that tetraploid cultivars of PRG had higher WSC concentrations than diploid cultivars. They found that even a tetraploid cultivar not specifically bred for WSC content (AberTorch) had greater WSC than a HSG diploid cultivar (AberDart), which was not the case in the current study where 2nHSNZ and 4nSt cultivars had similar WSC concentrations through all seasons. However, these findings contrast with the pattern observed by Robins and Lovatt (2016) and by Gilliland et al. (2002). In the former, diploid cultivars exhibited 8.20 g/kg DM higher WSC than the tetraploid cultivars as an average of five to seven harvests, while in the latter, no differences between cultivars were found as a mean of eight cuts, although differences were observed in one of those cuts. Both studies also compared different heading dates and found no differences in WSC among maturity classifications (early, intermediate, late). Similarly, Palladino et al. (2009), between late spring and early summer, found no difference in WSC and CP among heading dates (intermediate or late) or between diploid and tetraploid cultivars of PRG. Therefore, the superiority of 4nHSEU with respect to WSC concentration in early spring and autumn could not be attributed to the ploidy level or the heading date.

The other HSG tested here has its origin in a cross of PG158 and AberDart (IP Australia, 2014), a HSG bred in the UK by IGER (now IBERS, Aberystwyth University). Moreover, Expo (2nHSNZ) and AberDart have been classified as genetically similar cultivars given that both were in the same sub‐cluster, and in turn distant to the European cultivars sub‐cluster (Pembleton et al., 2016). Gilliland et al. (2002) compared WSC levels of 12 different PRG varieties under simulated grazing management and found that AberDart had the greatest WSC concentration (286 g/kg DM) while Calibra (4nHSEU cultivar) had a slightly lower concentration (278 g/kg DM), with both higher than the average (262 g/kg DM). Therefore, tests performed in Europe have shown that 4nHSEU and AberDart (the predecessor of the 2nHSNZ cultivar) express the HSG trait under those conditions.

A clear seasonal variation of WSC and CP was observed; WSC was lowest in early spring and increased until summer and then decreased to autumn. The opposite trend was observed for CP, with the maximum values in early spring and autumn, which is coincident with the annual pattern reported by Roche et al. (2009) and Loaiza, Balocchi, and Bertrand (2017) in their two‐leaf stage treatment. However, the tetraploid HSG differed from that general trend, containing similar WSC concentrations in August (early spring) and October (spring) and the maximum value in April (autumn).

### 4.2 Potential G × E interaction effect

Regarding the expression of the HSG trait in different environments, the 2nHSNZ cultivar has been tested in two sites in New Zealand (Manawatu – milder and Canterbury – colder). The WSC concentration and WSC to CP ratio were found to be lower in Manawatu (40°35′S) compared with Canterbury (43°38′S) (Easton et al., 2009), showing a G × E interaction (Rasmussen et al., 2009). Average WSC of 2nHSNZ cultivar at Manawatu was 217 g/kg DM (LSD = 20.9; *p* < 0.05), which is slightly higher than the average for this study (201 g/kg DM). However, Conaghan, O'Kiely, and O'Mara (2012), using AberDart (HSG) and Fennema (control) indicate that cultivar was the primary factor influencing WSC differences, being consistent across N application rates, years and locations. On the other hand, CP concentration found in this study for 2nHSNZ was slightly higher (216 g/kg DM) than that of Manawatu site (199 g/kg DM) which in turn was similar to a diploid standard cultivar (213 g/kg DM, LSD = 18.2; *p* < 0.05). Moreover, Easton et al. (2009) found that 2nHSNZ and AberDart had similar WSC, CP and WSC to CP ratio in the milder site, and both cultivars had lower WSC and higher CP concentrations compared at the colder site. Interestingly, either in the colder or the milder sites, both HSG cultivars showed lower CP and higher WSC concentrations and WSC to CP ratio than the tetraploid cultivar Banquet, the predecessor of the “4nSt” cultivar in this study. However, that trend was not observed in this study, where the 2nHSNZ and 4nSt cultivars did not differ in WSC in any season and CP concentration was similar between both cultivars (216 and 220 g/kg DM, respectively). Moreover, the lack of difference in WSC to CP ratio between cultivars groups (HSG vs. standard) found here contrasts with the pattern found by Easton et al. (2009) where the HSG cultivars (diploids and tetraploids) averaged 1.32 and the standard cultivars averaged 1.03. Our findings also contrast with the study of Turner, Donaghy, Pembleton, and Rawnsley (2015), where AberMagic maintained a mean WSC to CP ratio greater than Arrow, regardlesss of defoliation interval (1.5 and 3 leaf stage).

The potential G × E interaction that might be operating under the year of evaluation of the present study could lie on the requirement of a cold period to get the high sugar trait expressed (Parsons, Edwards, et al. (2011)) through its effect on the expression of the genes coding for the enzymes involved in the synthesis of fructans (Rasmussen, Xue, Newman, & Parsons, 2008). This requirement is probably more likely to be accomplish through the years under UK climate conditions, particularly in Aberystwyth, where these cultivars have been developed, where the long‐term average minimum and maximum temperatures are 6.7 and 13.5°C (Robins & Lovatt, 2016), which contrasts with the temperatures recorded in the year of evaluation of the present study (8.4 and 17.5°C, respectively). However, changes in mean temperature and interannual variation are expected to occur under a scenario of global warming (Boer, 2009), which might imply that the weather conditions required for the expression of the high sugar trait could be present in certain years. This highlights the need of long‐term studies to account for this potentially increased variability in environmental conditions.

Total annual DM production did not vary with cultivar nor agronomic management in the first year after sowing. Even though it is important to confirm that the elevation of WSC concentration of HSG does not occur at the expense of DM yield (Edwards, Parsons, & Rasmussen, 2007), some contrasting results have been reported between cultivars. For instance, Halling et al. (2004) reported that, compared to the standard cultivar Fannema, the HSG cultivar AberDart had lower DM yields at five sites and a significant greater DM yield at only one site, demonstrating a strong cultivar × site interaction, i.e., G  ×  E interaction. However, Bryant, Parsons, Rasmussen, and Edwards (2009) found no difference between HSG and standard cultivars, while Chapman, Muir, and Fvaille (2015) found a greater DM production of the standard cultivars over the HSG AberDart in some periods of a long‐term evaluation but those differences were not consistent through time. Moreover, Hume et al. (2010) found similar productivities between the HSG AberDart and the diploid standard cultivar Bronsyn across three years of evaluation in a warm site but significant differences were observed in a cooler site (both in New Zealand) during the same period. Moreover, even though the HSG and standard cultivar had similar DM yields in the first year, the HSG had greater productivity on the second and third year, and as an average of the whole period, again showing an interaction of the site of evaluation with the genotypes tested. On the other hand, Turner et al. (2015) found that high sugar cultivars (one from continental Europe and one from the UK) displayed the same or superior herbage DM yields compared with the control standard cultivar Arrow.

The lack of differences in DM production in the present study between HSG and standard cultivars could be either due to the effect of the particular year or site of evaluation, where the climate conditions prevent the cultivars from expressing their differences in productivity potential, or due to the lack of intrinsic differences among cultivars in terms of DM yield potentials. A longer‐term evaluation would be necessary to reach a conclusive answer. Interestingly, triplicating the N fertilization rate and defoliating more frequently, which decreased the WSC to CP ratio, did not ensure an increase in DM yield in the first year, which might indicate that lower N inputs with an appropriate defoliation regime can be as productive as the opposite strategy but with a positive effect on the energy to protein balance of the forage reaching the rumen.

### 4.3 Relative changes in nutrients concentration with increased WSC

According to Staerfl et al. (2012), increasing WSC in the plant cell will replace part of the fibre or the protein or even both fractions. In this study, considering simultaneously NDF and CP concentrations, a general trend to decrease both components when increasing WSC was observed. However, the effect on NDF concentration when increasing WSC concentration in leaves was not so consistent as CP reduction. Moreover, the tendency of the 4nHSEU cultivar to show a lower correlation coefficient between WSC and CP than the remaining cultivars is coincident with the higher values for WSC and CP concentrations of 4nHSEU in early spring and autumn compared with the remaining cultivars. It is relevant to analyse the relative changes in WSC, CP and NDF given that the effect on other plant nutrients when increasing WSC of grass may vary; therefore, the overall effect of a pasture with higher WSC may have different consequences on the animals grazing on that pasture. If the increase in WSC is at the expense of CP content, an improvement of NUE might be expected (Ellis et al., 2011; Staerfl et al., 2012). Likewise, increases in WSC that are accompanied by a reduction in NDF can result in greater DOMD and voluntary DM intake (Fraser, Fleming, Theobald, & Moorby, 2015). However, 4nHSEU had the greatest CP concentration among cultivars and that pattern was observed in early spring and autumn, when the WSC was also the highest, generating a WSC to CP ratio similar to the remaining cultivars. These finding would support the idea that selection for high WSC concentration in 4nHSEU cultivar was not at the expense of CP. Moreover, similarly to the study of Turner et al. (2015), CP concentration did not vary between the HSG cultivar AberMagic and control cultivar Arrow, supporting the study of Radojevic, Simpson, StJohn, and Humphreys (1994) who indicate that WSC concentration did not explain a significant proportion of the variance in N concentration (<1%).

As previously reported (Roche et al., 2009), ME was the lowest in summer and coincided with the greatest NDF and ADF concentrations. However, that inverse relationship was not observed in the remaining seasons. Moreover, Roche et al. (2009) found a similar pattern through the seasons between WSC and ME, which was not observed in this study and no clear relationship was found between these variables. Besides, these slight variations in ME content among cultivars and seasons (<0.40 MJ/kg DM) are not expected to have a biological impact. On the other hand, the positive and strong correlation found here between NDF and ADF was coincident with the value reported by Roche et al. (2009) for this relationship (*r* = 0.87). In this study, the general trend showed that the increase in WSC concentration in the grasses occurred at the expense of both CP and NDF, and both components contribute to the energy density of the diet (Taweel et al., 2005). Therefore, a higher WSC concentration does not necessarily mean a high‐energy density. According to Taweel et al. (2005), it may even mean lower energy density, as the WSC substitute part of the NDF and CP that are highly digestible in young and leafy grass.

According to Parsons, Edwards, et al. (2011), increases in WSC are inevitably associated with decreases in all other components, by the simple principle of “dilution.” Removing this “dilution” effect, Rasmussen et al. (2009) reveal that reductions in fibre concentration when WSC increased may not be a fundamental change in plant structural chemistry, and in several circumstances, an increment in WSC concentration of HSG is associated with either no change or even a small increase in NDF, and only CP appeared to be reduced (Parsons, Edwards, et al. 2011). This non‐consistent relationship between NDF and WSC with increased WSC concentration is similar to the trend observed in this study with a positive weak single regression coefficient between these components and a negative and variable multiple regression coefficient for NDF when considered together with CP as a predictor of WSC concentration. Moreover, changes in CP content with increased fibre fractions seem to be more consistent under a more frequent defoliation regime.

### 4.4 WSC to CP ratio

With a target WSC to CP ratio of 0.70–0.75 (Edwards, Parsons, & Rasmussen, 2007; Pacheco, Burke, & Cosgrove, 2009), which is the level beyond urinary N is reduced by increasing WSC, it is observed that neither cultivar nor management could increase that ratio above the critical level (ranged between 0.39 and 0.62, data not shown) in early spring. Similar values were reported by Loaiza, Balocchi, and Bertrand (2017) in early spring for a PRG pasture managed at 2 and 3 leaves per tiller and with different N fertilization rates (from 0 to 450 kg^−1 ^ha^−1 ^year^−1^) who reported WSC to CP ratio between 0.26 and 0.57 for pastures defoliated at 2 leaves per tiller and 300 kg N ha^−1 ^year^−1^ and at 3 leaves per tiller and 75 kg N ha^−1 ^year^−1^, respectively. Interestingly, all combinations of cultivar by management surpassed 0.70 in spring and autumn. Particularly, favourable management increased on average 0.33 units that ratio during those seasons, which could imply circa 5 percentage points in NUE (g milk protein N per 100 g N ingested) for dairy cows grazing PRG pastures under this management (Pacheco et al., 2009). Moreover, Edwards, Parsons, & Rasmussen, 2007 and Parsons, Edwards, et al. (2011) suggested that to harness the benefits of HSGs, in improving NUE, depended on a sufficient elevation of the WSC to CP ratio from 0.6 to 1.2–1.5. In this study, this range of values was only achieved under the favourable management in spring by the 4nSt cultivar (1.48) and in autumn by both tetraploid cultivars (1.26–1.28). This could imply that the improvement in the balance between energy and N in the rumen by only using HSG cultivars is not assured.

### 4.5 Effect of pasture management

Given that appropriate time for regrowth is required to recover from defoliation and the accumulation of WSC reserves; WSC concentrations in PRG increase until a maximum measured at the 3‐leaf stage (Loaiza, Balocchi, and Bertrand 2017; Turner et al., 2015), and management alone can be used in some circumstances to modify diet WSC to CP ratio (Parsons, Rowarth, & Rasmussen, 2011). Favourable management (longer regrowth time and lower N fertilizer rate) notably increased overall WSC and decreased CP, leading to an improvement in the WSC to CP ratio, compared with an unfavourable management (shorter regrowth time and higher N fertilizer rate). However, this pattern was not consistent among seasons for all variables. Our findings show that it is not possible to increase the WSC to CP ratio in early spring above 0.7, and this is due to the extremely high CP concentration at this critical time of the year (above 29%).

Rasmussen et al. (2009) proposed that there is an interplay between temperature and regrowth duration, exposing a possible conflict given that low temperatures *per se* increase WSC concentration in leaves, but following a defoliation, warmer temperatures may stimulate faster recovery of leaf area during regrowth, leading to higher WSC levels. The highest WSC observed in summer for the favourable management is coincident with the joint effect of longer regrowth periods and warmer environments (Parsons et al., 2004). These authors also observed that at warm days and low night temperatures (20°C/10°C), the HSGs sustained significantly higher WSC than control cultivars. Similar maximum and minimum temperate was recorded in this study for autumn sampling (17.8°C/9.8°C), and coincidently one HSG cultivar, the tetraploid originated in Europe (4nHSEU), showed a greater value than the remaining cultivars. However, the diploid HSG cultivar bred in New Zealand (2nHSNZ) did not show a positive effect of the temperature regime on the WSC concentration.

### 4.6 Potential for improving animal performance through HSG cultivars

High sugar cultivars development has increased concentrations of WSC to circa 20–40% of DM, compared to conventional commercial varieties (10–15% of DM; Lee et al., 2001). In this study, even though no combination of cultivar by management reached WSC concentrations >200 g/kg DM in early spring (critical time of the year regarding WSC concentration and WSC to CP ratio), the tetraploid HSG cultivar showed a high WSC concentration in autumn (the other crucial season) averaging 266 g/kg DM and representing 55.1 g/kg DM higher than the remaining cultivars. The aim of IGER when developing HSG was to increase WSC concentration beyond that of a standard cultivar to a minimum expected to lead to a significant change in animal performance. Increases in live weight in lamb have been achieved when the differential in grass WSC concentration was approximately 40 to 50 g/kg DM (Lee et al., 2001). However, for high‐N growing situations, which could be the case of the 4nHSEU cultivar, the calculation suggests that increases of more than 100 g/kg DM in WSC concentration would be required (Parsons, Rowarth, & Rasmussen, 2011).

### 4.7 Implications of our findings to the farming community and breeding industry

As HSG cultivars have not shown a consistent expression of the “high sugar” trait, it will be difficult for farmers to trust in that expression only for the fact of buying a cultivar marketed as high sugar. For farmers, a better way to ensure a higher WSC concentration should be by means of pasture management, in terms of grazing criteria and fertilizer use as expressed here.

It will be a challenge for plant breeders in terms of the way cultivars (or traits) are evaluated. The evaluation of traits of interest must be supported by the agronomic research of the G  ×  E interaction (Lee, Matthew, Thom, & Chapman, 2012) in multiple environments. In this aspect, Robins and Lovatt (2016) evaluating six environments suggest that environments with higher temperatures will result in lower levels of WSC and that water stress environments are a way to express the genetic variation underlying WSC.

## 5 CONCLUSIONS

Within a 12‐month study at one site under the temperate climate and soil conditions of southern Chile, perennial ryegrass cultivars have not shown a consistent expression of the “high sugar” trait in the establishment year, given that only the European HSG cultivar showed some superiority during the critical seasons, i.e., early spring and autumn. This is not in agreement with our hypothesis that the diploid HSG cultivar developed in New Zealand may have greater potential to express the trait given the similarities in climate conditions between New Zealand and southern Chile. However, the European HSG cultivar also had the highest CP concentration, determining no differences among cultivars regarding the WSC to CP ratio. All cultivars were above 1.0 on average between spring (October) and autumn (April), and no combination of cultivar and management could improve the ratio in early spring (August) to reach the critical level of 0.7–0.75. The agronomic management, i.e., defoliation frequency and N fertilization rate, shows a more consistent effect on modifying WSC and CP concentration among the seasons than cultivar. Our findings support the idea that a G × E interaction might be operating in the temperate climate conditions that prevent the HSG trait being expressed consistently although a longer‐term study would be required to confirm this.

## CONFLICT OF INTEREST

The authors certify that they have NO affiliations with or involvement in any organization or entity with any financial interest, or non‐financial interest in the subject matter or materials discussed in this manuscript.
